# Investigations Into the Suitability of Bacterial Suspensions as Biological Indicators for Low-Energy Electron Irradiation

**DOI:** 10.3389/fimmu.2022.814767

**Published:** 2022-04-29

**Authors:** Simone Schopf, Gaby Gotzmann, Marleen Dietze, Stephanie Gerschke, Lysann Kenner, Ulla König

**Affiliations:** Division Medical and Biotechnological Applications, Fraunhofer Institute for Organic Electronics, Electron Beam and Plasma Technology, Dresden, Germany

**Keywords:** bacteria, dosimetry, liquid, inactivation, impedance spectroscopy

## Abstract

Low-energy electron irradiation is an emerging alternative technology for attenuated or complete pathogen inactivation with respect to medical, biotechnological, and pharmaceutical applications. Pathogen inactivation by ionizing radiation depends mainly on the absorbed electron dose. In low-energy electron irradiation processes, determination of the absorbed electron dose is challenging due to the limited, material-dependent penetration depth of the accelerated electrons into the matter. In general, there are established dosimetry systems to evaluate the absorbed dose under dry irradiation conditions. However, there is no system for precise dose monitoring of low-energy irradiation processes in liquids or suspensions so far. Therefore, in this study three different bacterial species were investigated as biological dose indicators, especially in the range of low doses (< 6.5 kGy) in aqueous solutions or suspensions. *Escherichia coli*, *Bacillus subtilis*, *and Staphylococcus warneri* were comparatively evaluated for their suitability as biological dose indicators. Thin homogeneous films of the respective bacterial suspensions were irradiated with increasing doses of low-energy accelerated electrons. The average absorbed dose was determined using a colorimetric dosimeter based on a tetrazolium salt solution. The maximum and minimum absorbed doses were measured with a referenced film dosimeter. Subsequently, the inactivation kinetics was determined in terms of inactivation curves and D_10_ values. Thus, the minimum inactivation dose of bacterial growth was assessed for *E. coli* and *S. warneri*. The effect of irradiation with low-energy accelerated electrons on the growth behavior and activity of the bacteria was studied in more detail using impedance spectroscopy. With increasing irradiation doses growth was delayed.

## Introduction

The inactivation of microorganisms is a critical step in many aspects of biomedical research, in biotechnological production processes, and in healthcare facilities. Sterilization is a validated process that destroys or eliminates all forms of microbial life, leaving a product free from viable microorganisms. Various physical or chemical processes are used to achieve sterility or to inactivate microorganisms. Amongst physical treatment, ionizing radiation with gamma-rays, X-rays, and high-energy accelerated electrons has been used as technology for pharma applications and to sterilize medical products or pasteurize food (1–4). In recent years, electron beam accelerators have emerged as feasible alternative for industrial processing. Sterilization using accelerated electrons is an accepted technology that meets the requirements of international standards according to (5). For radiation sterilization a minimum dose of 25 kGy is required to achieve sufficient sterilization efficiency. Consequently, microorganisms such as bacteria, viruses, and protozoa can be efficiently inactivated with technologies using accelerated electrons (6–8).

Depending on the kinetic energy of the electrons, electron beam technology can be distinguished either in high-, middle-, or low-energy accelerated electron irradiation (LEEI; ≤ 300 keV) (9). The penetration depth of the electrons is determined by their kinetic energy, by the density, and by the thickness of the treated material. The higher the kinetic energy and the lower the density of the matter, the higher the range of electrons in the material (10). Electrons with high kinetic energy can penetrate products up to several centimeter whereas the penetration depth of low-energy accelerated electrons is limited to a few hundred micrometer (11). Thus, the low penetration depth is a major challenge when using LEEI (12), especially for liquid processing systems. Consequently, the liquid, e.g. a pathogen-containing suspension, has to be processed as a thin film of several microns to ensure homogeneous irradiation through the complete liquid film.

When low-energy accelerated electrons collide with matter their kinetic energy is transferred through physical interactions to excite molecules. This leads to the formation of highly reactive free radicals, such as hydroxyl radicals. These hydroxyl radicals can initiate a cascade of chemical chain reactions leading to the breakdown of structural and functional biomolecules. The most important target for the action of ionizing radiation in the cell is the DNA molecule. The DNA destruction leads to irradiation damage of the cells (13). Furthermore, the inactivation of pathogens can also be attributed to the degradation of nucleic acids, either by direct interaction or indirectly through the radiolysis of water within the cell (4). Hydroxyl radicals are thought to be responsible for 80-90 % of total DNA damage (14).

High-energy electron accelerators generate a large amount of Bremsstrahlung (X-ray radiation). Therefore, these irradiation facilities must be equipped with complex shielding constructions to protect both the personnel and the environment. This makes direct integration of HEEI technologies into pharmaceutical production facilities challenging. In contrast, LEEI technology generates only a low quantity of X-ray radiation, which minimizes undesired side effects and allows for compact radiation protection. In addition, LEEI is a chemical-free, fast process with high overall energy efficiency (11, 15). Previously, LEEI has been shown to be an emerging technology for the development and production of inactivated vaccines (16).

The benefits of LEEI technology are faced with the challenge of determining the absorbed dose in irradiated liquids, especially in the range below 6.5 kGy. In general, there are established dose indicators for measuring the dose under dry irradiation conditions (1, 17). There is a whole range of approved, excellent dosimeters, e.g. the reference standard alanine dosimeter (18), which can be used to calibrate other dosimeters, and the Risø B3 radiochromic film dosimeter for routine dose measurements (19).

However, the radiation-induced response from some routine dosimeters is not stable and changes with time after irradiation (20). Existing film dosimeter systems are susceptible to environmental influences, such as air, humidity, oxygen content in the atmosphere, surrounding temperature, or UV radiation from sun light (21). In addition, the response can vary by up to 30-40 % if the dosimeters have been irradiated under extremely dry or humid conditions, which has a vast impact on the result. For LEEI applications, it is recommended to store and irradiate the film dosimeters under controlled and well-defined conditions (21). A liquid dosimeter currently in use is based on a dye solution that changes its spectral properties after irradiation with low-energy accelerated electrons (8). However, this liquid dosimeter is only reliable for doses above 6.5 kGy. Hence, there is a necessity for a liquid routine dosimeter for low doses of low-energy accelerated electrons.

A biological dose indicator system based on various bacteria (hereafter referred to as bio-dosimeter) could potentially improve the quantification of the dose in liquids after irradiation with low-energy accelerated electrons. The underlying assumption is that bacteria lose their ability to proliferate after being exposed to a certain dose of low-energy accelerated electrons. Consequently, the response of the dosimeter should remain constant and bias due to improper storage of the bio-dosimeter should be avoided.

In this study, three different non-pathogenic bacteria from different taxa and with different physiological characteristics were studied for their suitability as biological dose indicators for LEEI. *Escherichia coli* served as a Gram-negative model organism. *Staphylococcus warneri* and vegetative cells of *Bacillus subtilis* served as Gram-positive representatives. The objective was to characterize the inactivation kinetics of each bacterium and to investigate the impact of LEEI on growth and activity by impedance spectroscopy.

## Material and Methods

### Strains and Culture Conditions

All bacterial strains used in this study were obtained from the German Collection of Microorganisms and Cell Cultures DSMZ and kept as stock cultures at -20°C. *E. coli* K12 (DSM 498) was cultivated in Standard Nutrient Broth I and vegetative cells of *B. subtilis* (DSM 10) in LB-broth (both Carl Roth GmbH + Co. KG) at 37°C and 125 rpm shaking. *S. warneri* (DSM 20036) usually appears in conglomerates. To avoid agglomeration of the bacterial cells, CASO-Bouillon (Carl Roth GmbH + Co. KG) was used for cultivation at 37°C and 125 rpm overnight. Prior to each irradiation experiment a fresh overnight culture was grown. To ensure equivalent cell densities, the freshly grown pre-cultures were enumerated with a Neubauer improved counting chamber and diluted to approx. 10^6^ bacteria/ml.

**Figure 1:**
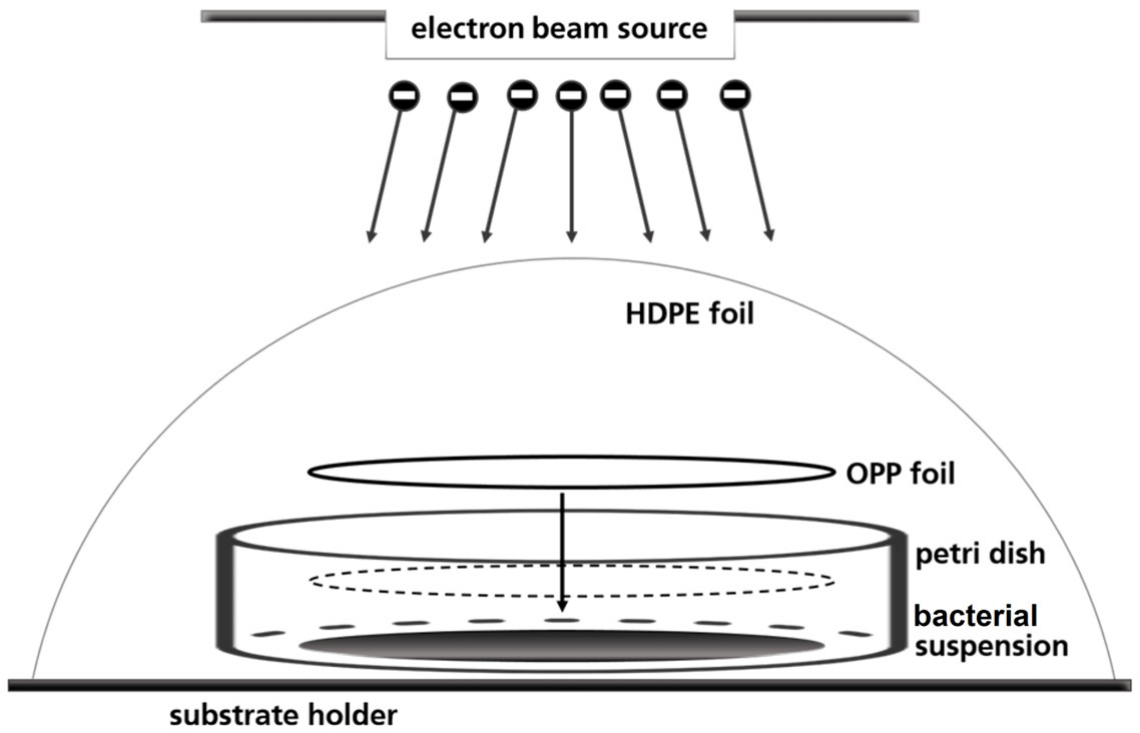
Schematic illustration of the OPP-system used for the irradiation of the bacterial suspensions.

A petri dish-based setup was used to irradiate the bacterial suspensions, in which the liquid droplet was covered with a round foil of oriented polypropylene (OPP) to create a thin homogeneous liquid film of 80 μm height (**Figure 1**). Prior to use, the OPP foils were disinfected with 70 % ethanol (v/v) for 15 minutes. 57 µl bacterial suspension was pipetted in three sterile petri dishes in a laminar flow work bench. The fourth petri dish served for routine dosimetry as described below. Briefly, a piece of dosimeter film was fixed in the center of the petri dishes and covered with OPP-foil. The so prepared petri dishes were fixed on one sample holder and covered with high-density polyethylene (HDPE) foil.

The low-energy accelerated electron plant REAMODE (“Reactive Modification with Electrons”) with a 200 keV electron beam (KeVac System, Linac Technologies, Orsay, France, 200 kV) was used for irradiation. The conveyor speed was 140 mm/s and the distance of the beam exit window to the specimens was 35 mm. The applied current (0.1-0.85 mA) was used to adjust the intended absorbed dose in a range from 0.1 to 3.5 kGy.

### Counting of Viable Cells After Irradiation

30 µl of bacterial suspension was immediately recovered from the petri dish and plated onto respective agar plates to determine the number of colony forming units (CFU). Plates were incubated for 24-48 h at 37°C. To determine the CFU/ml, the mean values of the visible colonies were calculated from three to nine agar plates.

For solely qualitative analysis of bacterial growth after irradiation, 5 ml of sterile nutrition broth was added to the remaining bacterial suspension in the petri dish. The so prepared petri dishes were incubated at the optimum growth temperature for 7 days to monitor the growth of bacteria *via* turbidity.

### Routine Measurement of Absorbed Doses and Depth Dose Distribution

**Figure 2:**
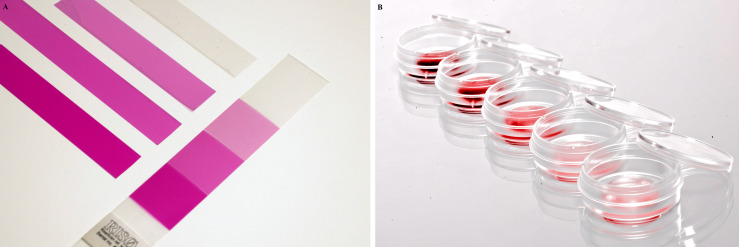
Film and liquid dosimeter. **(A)** Risø B3 film dosimeter. **(B)** TTC-based liquid dosimeter.

The absorbed dose was routinely measured with radiochromic films (**Figure 2A**; Risø B3 dosimeter, Risø High Dose Reference Laboratory, Roskilde, Denmark). The dosimeter films were incubated at 60°C for 8 min after irradiation. The absorbed dose was measured by quantifying the color change at 554 nm using special software with a calibration (RisøScan-System). Irradiation with low-energy accelerated electrons was accompanied by a dose gradient across the thickness of the irradiated dosimeter foil. Therefore, the dose measured with Risø B3 was corrected to Dµ, which corresponds to the absorbed dose in the first micrometer of the absorbing medium (12).

The dose gradient in the irradiated liquid was simulated by a stack of Risø B3 films (depth dose distribution). The total volume of bacterial suspension in the petri dish (57 μl) corresponded to a liquid height of 80 µm. Based on the density of a Risø B3 film (ρ = 1.12 g/cm^3^) an equivalent to the density of water was calculated (ρ = 1.0 g/cm^3^). Thus, a layer of Risø B3 (18 μm) corresponded to a liquid layer height of 20.1 μm. Consequently, a stack of 5 Risø B3 films was required to simulate a liquid height of 80 µm. Air bubbles between the dosimeter films were avoided.

After irradiation, the maximum irradiation dose was obtained from the response of the dosimeter on the top of the stack. The average dose was calculated from the mean value of 4 dosimeter films. The minimum dose was given as an average of the second last and the bottom dosimeter film. In order to measure the mean irradiation dose of the liquid, a dosimeter based on a solution of 2,3,5-triphenyltetrazolium chloride (TTC; 0.2 % (w/v)) in water was used (**Figure 2B**). The TTC was reduced to red-colored formazan upon irradiation. The absorption was measured in transparent 96-well plates at a wavelength of 485 nm (Tecan infinite M200, Tecan Group Ltd.) (8). The TTC was calibrated using the Risø B3 dosimeter. The calibration procedure will be described elsewhere.

### Determination of D_10_ Values

The D_10_ value is defined as the dose required to reduce the population by 1 log or decrease it by 90 %. It is calculated from the negative reciprocal of the slope of the regression line that is produced by plotting the number of colony forming units against the absorbed dose. The mean D_10_ value (n = 3) was calculated for each of the three species. From the simulation of the depth dose distribution the maximum and the minimum absorbed doses were measured and calculated. The theoretical inactivation dose was given as the radiation dose that reduced the initial population by 10^6^ bacteria/ml or by a 6 log reduction. To calculate the theoretical inactivation dose for each species the D_10_ value was multiplied by 6.

### Impedance Spectroscopy

By impedance spectroscopy, the measured change in the electrical conductivity allows the qualitative and quantitative tracing of microorganisms due to the analysis of their microbial activity. Uncharged or weekly charged components of the growth medium are metabolized into smaller charged components by the bacteria, which changes the electrical conductivity of the growth medium and thus the impedance signal. For the measurements carried out in this study, the BacTrac 4100^®^ system (Sy-Lab, Austria) was used with the electrode impedance or E-value (E %). The impedance curve showed the relative change of the E-value as a function of time measured in intervals of 10 minutes.

For *E. coli*, 5970 µl nutrition broth was transferred to an impedance cell. 30 µl of the respective bacterial suspension was added. The suspensions were collected from the petri dish (OPP-system) after irradiation with 0.7, 1.4, 2.1, and 2.8 kGy, respectively. The positive controls (referred to as 0 kGy samples) were not irradiated but handled identically to the irradiated samples. As negative controls sterile growth medium was used.

In an optimized procedure for *S. warneri* and *B. subtilis*, 5700 µl growth medium (CASO bouillon or LB broth) was transferred to an impedance cell. To the (irradiated) bacterial suspension in the petri dish, 513 µl of the respective growth medium was added to the edge of the OPP foil. The petri dish was shaken at 100 rpm for 2 min. 300 µl of the diluted bacterial suspension were rinsed 2-3 times to achieve maximum recovery of bacteria. The collected bacterial suspension was transferred to the impedance cell. All impedance analyses were performed at 37°C.

### Statistical Analysis

Data in the figures are given as mean values, and, if indicated, with ± standard deviation (SD). The standard deviation was calculated by the standard error of the arithmetic mean using the MiniTab 20 statistical software. To determine the D_10_ values regression analysis was performed with MiniTab 20 or Excel 2016.

For impedance spectroscopy, all measurements were carried out in triplicates within one series of experiments and additionally at three independent time points (n = 9). Positive controls and negative controls were carried out in triplicates, too.

## Results

### Dosimetry

**Figure 3:**
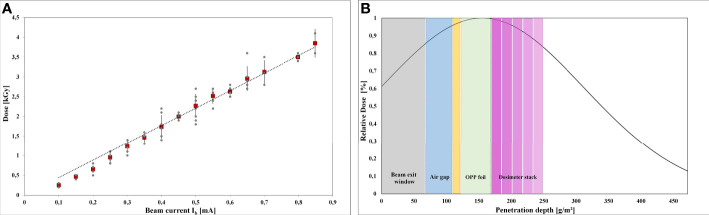
Dose measurements and depth dose distribution: **(A)** Linear correlation between the adjusted beam current and the absorbed dose in the intended dose range from 0 to 3.5 kGy. The red rectangles show the mean values. **(B)** Schematic illustration the depth dose distribution in the OPP-system, simulated by a stack of 5 dosimeter films.

Accurate dose measurement was critical for reliable results **(** **Figure 3**). The intended dose was adjusted by the beam current intensity. The applicable dose range of the Risø B3 film dosimeter is reported to range from 5 to 100 kGy. Since the doses for LEEI inactivation of the used microorganisms are below 5 kGy, which is the Risø B3 lower limit, measuring fluctuations in the dosimeter response can occur. Therefore, dose values from three independent LEEI experiments were plotted against the beam current (**Figure 3A**). There was a linear correlation between beam current and absorbed dose with an R^2^ value of 0.989 and a standard deviation of about ± 4 % for the applied doses.

Due to the low penetration depth of the low-energy accelerated electrons, a dose gradient within the bacterial suspension was expected during LEEI. Therefore, experiments were carried out to simulate the depth dose distribution across the liquid using a stack of Risø B3 films as reference dosimeter (**Figure 3B**). The measured and calculated maximum, mean, and minimum dose values are given in **Table 1**. From the maximum absorbed dose (corresponding to 100 %), a relative minimum dose of 84.4 % was derived. This relative percentage value served as calculation basis for the minimum D_10_ value.

**Table 1:** Depth dose profile derived from Risø B3 films stacked within the OPP-system.

Nominal dose [kGy]	Absorbed max. dose[kGy]*	Absorbed mean dose[kGy]**	Absorbed min dose[kGy]***	% relative to max. dose	Absorbed mean dose in liquid by TTC [kGy]
20	20.8	20.0	17.9	85.8	20.5
25	24.7	23.4	20.4	82.6	27.1
30	31.5	29.8	26.7	84.8	27.9

*Derived from the uppermost dosimeter film, **the mean value from 4 films, and ***the mean value from the second-last and bottom film. The mean dose determined with the TTC liquid dosimeter is given in the right column.

As colorimetric dosimeter for liquids, the TTC dye indicator was used. However, with the colorimetric liquid dosimeter, only the average dose related to the total volume of the liquid could be determined. The liquid dosimeter did neither provide information on the dose gradient nor on the maximum and minimum absorbed dose. However, the mean dose values measured with the TTC liquid dosimeter were in good agreement with the mean dose values calculated for the Risø B3 film stack (**Table 1**).

### Inactivation Curves and D_10_ Values

**Figure 4:**
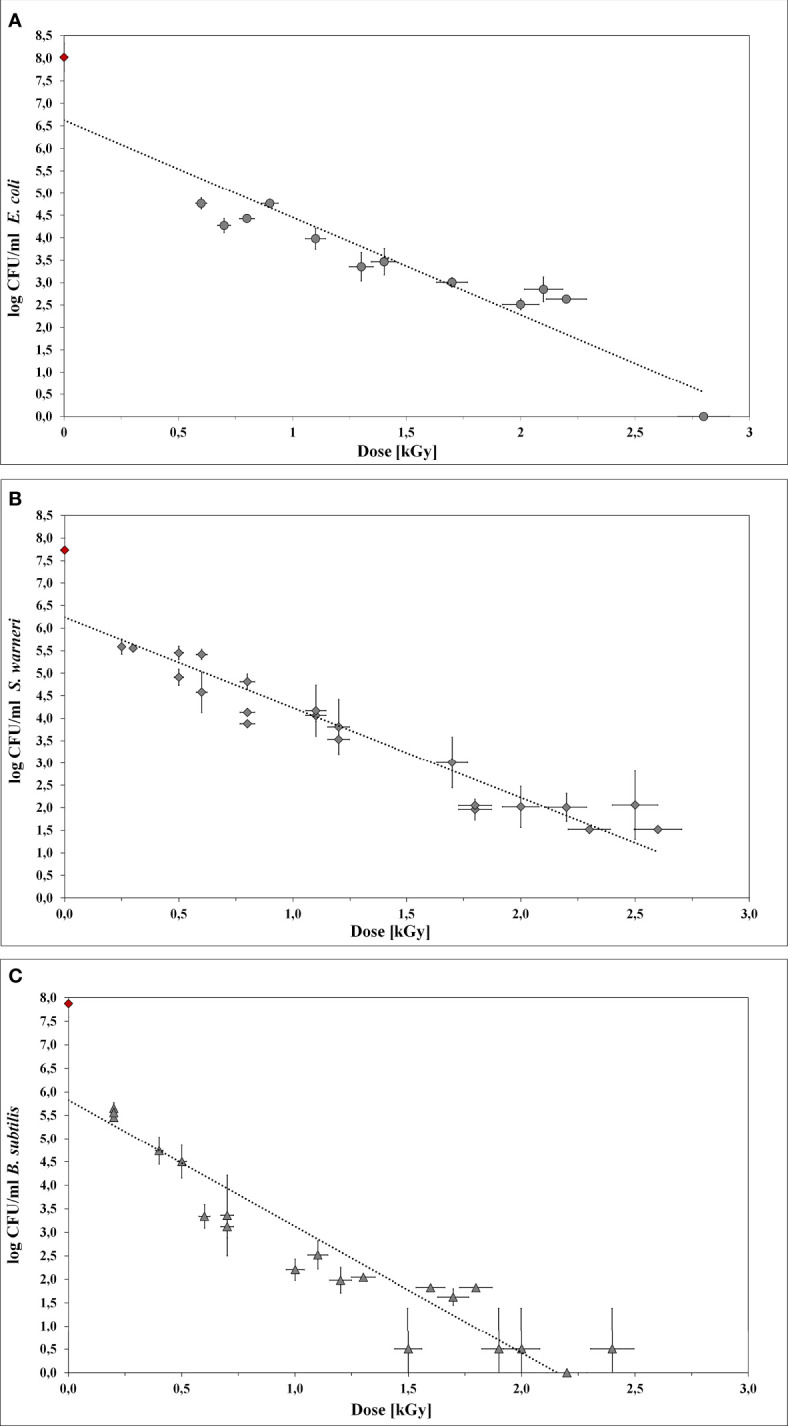
Inactivation curves using LEEI. Each curve represents the mean values of at least three irradiated bacterial suspensions. The initial cell number of the pre-culture (indicated as red rhombus) was approx. 10^8^ bacteria/ml. The starting total cell number for the experiments was 10^6^ bacteria/ml. **(A)** *E coli.* **(B)** *S. warneri*. **(C)** Vegetative cells of *B. subtilis*.

LEEI experiments were carried out to determine the dose values required for the inactivation of *E. coli*, *S. warneri*, and *B. subtilis* **(** **Table 2****)**. Initially, 10^6^ bacteria/ml were irradiated with increasing doses up to 3.5 kGy. Upon irradiation, the bacteria were plated on agar-plates to determine the number of surviving cells. The number of CFU was plotted as a function of the dose to obtain the D_10_ values (**Figure 4**). In all cases, the counts of CFU decreased linearly as the dose increased. The R^2^ values from the regression lines were 0.872, 0.903, and 0.871 for *E. coli*, *S. warneri*, and *B. subtilis*, respectively.

**Table 2:** D_10_ values and the maximum theoretically and experimentally determined inactivation dose values for *E. coli*, *S. warneri*, and *B. subtilis*.

Bacterium	D-10max [kGy]	D-10min [kGy]	Maximum in activation dose (theoretical) [kGy]	Maximum in activation dose (experimentally) [kGy]
*E. coli*	0.46	0.39	2.76	2.80
*S. warneri*	0.50	0.42	2.99	2.80
*B. subtilis*	0.37	0.31	2.2	n. d.

N. d., not determined.

Irradiation with low-energy accelerated electrons was associated with dose gradient across the bacterial suspension. Therefore, the maximum and minimum absorbed dose was taken into account.

For *E. coli* (**Figure 4A**) the application of 2.8 kGy reduced the initial cell density of 10^6^ cells/ml to 0 CFU. Additionally, no bacterial growth occurred after sterile growth medium was added to the petri dishes for recultivation of potentially surviving or recovering bacteria. Since the media did not become turbid, the presence of multiplying *E. coli* cells was excluded. Thus, 2.8 kGy was experimentally determined as the required dose to inhibit growth. This was in good accordance with the calculated maximum inactivation dose (2.76 kGy), which was derived from the maximum D_10_ value multiplied by 6. The calculated minimum inactivation dose was approximately 2.4 kGy. Furthermore, the maximum and minimum dose to obtain a 1 log reduction for *E. coli* was calculated. The corresponding D_10_ values were 0.46 kGy (derived from the negative reciprocal of the slop from the inactivation line) and 0.39 kGy (84.4 % of the maximum applied dose), respectively.

*S. warneri* did neither grow on agar-plates nor after adding sterile growth medium to the petri dishes after irradiation with 2.8 kGy. The maximum and minimum inactivation dose was 3.0 kGy and 2.9 kGy, and the corresponding maximum and minimum D_10_ values 0.50 kGy and 0.42 kGy (**Figure 4B**).

Vegetative cells of *B. subtilis* were, according to the calculated doses, supposed to be inhibited in growth in a range from 1.9 kGy to 2.2 kGy, resulting in D_10_ values from 0.31 kGy to 0.37 kGy, respectively (**Figure 4C**). However, the *B. subtilis* inhibition could not clearly be achieved because the turbidimetric test results even at higher doses were not reproducible. The presence of surviving and multiplying *B. subtilis* cells was indicated.

### Characterization of Growth *via* Impedance Spectroscopy

**Figure 5:**
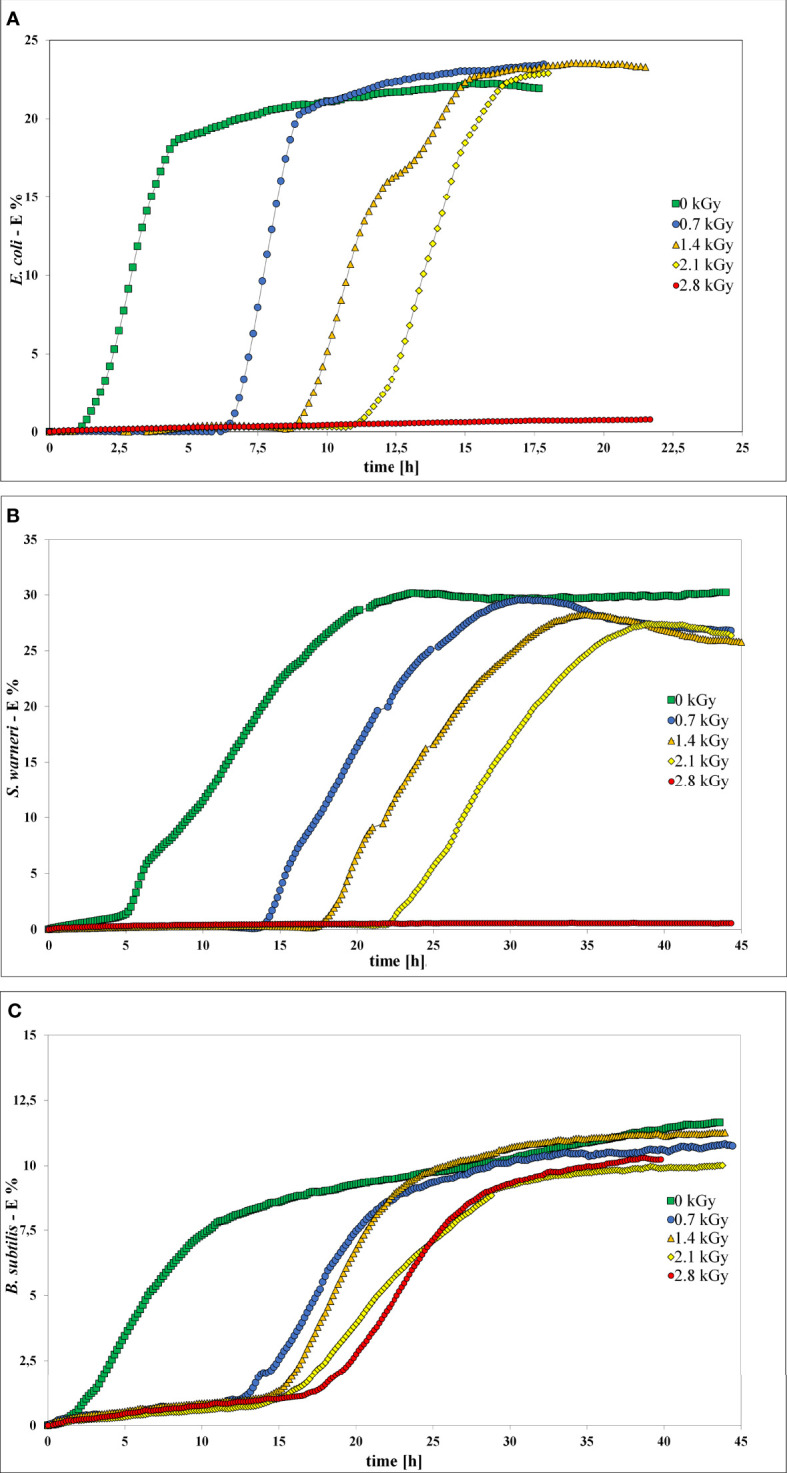
Impedance spectra without (0 kGy, green rectangles) and after irradiation with at 0.7 kGy (blue circles), 1.4 kGy (orange triangles), 2.1 kGy (yellow rhombs), and 2.8 kGy (red rectangles). **(A)** *E*. *coli*. **(B)** *S. warneri*. **(C)** Vegetative cells of *B. subtilis*.

Impedance measurement was used to analyze the influence of LEEI on growth and thus metabolic activity of *E. coli*, *S. warneri*, and *B. subtilis* (**Figure 5**). Bacterial suspensions were collected after irradiation with 0.7, 1.4, 2.1, and 2.8 kGy, respectively. Samples containing bacterial suspensions, which were handled identically but not irradiated served as controls (0 kGy). As negative control sterile non-inoculated liquid growth medium was used. Those controls did not produce any measurable change in the electrical impedance signal. When bacterial growth occurred, the resulting impedance spectra showed a sigmoidal trend. Positive controls were carried out using a freshly grown pre-culture, which was not handled in the OPP-system. The impedance signals recorded from those controls were similar to the 0 kGy control samples (data not shown). After irradiation of the bacteria with 0.7 kG and 1.4 kGy, growth of *E. coli* and *S. warneri* was measurable in terms of an impedance signal in all samples. Irradiation of *E. coli* and *S. warneri* with 2.1 kGy resulted in a transition state in which two of nine samples were already inactivated, whereas the remaining ones produced an impedance signal **(** **Figures 5A, B****)**. After irradiation with 2.8 kGy, basically complete inactivation was achieved, i.e. the impedance signals did not increase.

For *B. subtilis*, impedance analysis revealed a different picture (**Figure 5C**). Complete inactivation could not be reproducibly achieved and growth occurred in some samples even after irradiation with 2.8 kGy was applied.

In general, irradiation with increasing doses from 0.7 to 2.1 kGy resulted in a prolongation of the lag phase. The entry into the exponential phase was delayed, as also summarized in **Table 3**. This finding was very pronounced in *E. coli* and *S. warneri*, whereas it was less evident in *B. subtilis*. However, in all three species studied, the slope of the curve in the exponential growth phase was not significantly affected.

**Table 3:** Influence of increasing dose values on the start of the exponential phase (start log-phase) given as mean value with the respective standard deviation (SD).

Applied Dose [kGy]	*E. coli*			*S. warneri*			*B. subtilis*		
	Start log-phase [h]	SD	Total IA	Start log-phase [h]	SD	Total IA	Start log-phase [h]	SD	Total IA
0	1.7	± 0.5	0	5.6	± 0.4	0	2.8	± 0.8	0
0.7	6.4	± 0.4	0	14.5	± 0.3	0	12.6	± 3.2	0
1.4	10.6	± 2.5	0	18.5	± 0.6	0	13.9	± 2.1	0
2.1	11.5	± 2.1	2	22.6	± 1.9	2	15.6	± 2.2	1
2.8	14.3	± 3.5	7	23.0	n. d.	8	18	± 5.8	3

The absolute number of complete inactivated bacterial samples is given (total IA). N. d., not determined.

## Discussion

It was previously shown that LEEI can successfully be used to inactivate a number of pathogens, such as influenza (H3N8), Equid herpesvirus 1, respiratory syncytial virus (RSV), and bacteria (6, 16, 22). However, up to now, no liquid dosimeter is available for low-energy accelerated electrons and doses below 6.5 kGy. To overcome this issue, the suitability of different bacteria as bio-dosimeter based on their radiation susceptibility was investigated. Therefore, *E. coli*, *S. warneri*, and *B. subtilis* (as vegetative cells) were irradiated with doses < 6.5 kGy to determine the inactivation dose, the D_10_ value, and to investigate the effect of LEEI on the growth behavior in more detail *via* impedance spectroscopy.

### Dosimetry

The low penetration depth of LEEI technologies into matter is challenging, when using this technology for biological processes in liquids or suspensions. Therefore, the irradiation process with low-energy accelerated electrons was carried out in a petri dish system using a cover foil of oriented polypropylene (OPP) to achieve a thin homogenous liquid layer (approx. 80 μm) with low dose gradient. The depth dose distribution was determined using the Risø B3 film dosimeter as calibrated reference dosimeter. A transfer of the mean dose measurement data into liquid systems was carried out by using a colorimetric TTC-based liquid dosimeter.

With the implementation of a routine procedure that strictly considers the time of evaluation after irradiation, the TTC-based liquid dosimeter is usable as stable dosimeter for LEEI in a dose range between 6.5 and 40 kGy (8). However, it does not provide optimal results at lower doses. There is an estimated uncertainty range of 11.4 %, especially since the applied the low doses used in this study were outside of the TTC calibration limits.

### Inactivation Curves and Minimal Inhibitory Dose

Complete bacterial inactivation, i.e. the inhibition of growth, of defined titers of bacterial cells by lethal electron doses is feasible based on the knowledge of the target organism’s D_10_ value (23). Since the applied sources of ionizing radiation and the irradiation conditions differ between studies, a direct comparison is challenging (24). There are several studies dealing with the inactivation of other bacteria by accelerated electrons as source of ionizing radiation, i.e. *Rodentibacter pneumotropicus* (22), *Salmonella enterica* serovar Typhimurium (25), or *Listeria monocytogenes* (26). Using LEEI, a dose of 5 kGy was sufficient to inactivate *E. coli* DH5α from a pre-culture with an OD600 of 3.0 in PBS, reproducibly. Inactivation kinetics (log reduction and D_10_) were not presented in the previous study using the petri dish system for irradiation (16). In the study shown here, the number of colony forming *E. coli* decreased linearly with increasing doses of low-energy accelerated electrons up to a dose of 2.8 kGy, which was sufficient to inhibit bacterial growth.

In a previously conducted study using high-energy accelerated electrons a dose of 7 kGy was required to prevent a defined titer of *E. coli* K12 from multiplication. It could be shown that the *E. coli* cells stayed metabolically active up to 9 days after irradiation, had intact membranes, and still supported propagation of bacteriophages (27). Another study investigated electron beam technology for food preservation and revealed that a dose of 1.0 kGy reduced the growth of *E coli* in nutrient broth by 3-4 log units (D_10_ = 0.27 kGy). No bacteria were detectable after an irradiation with 2 kGy of high-energy accelerated electrons (10 MeV). However, when grown on meat a dose of 1 kGy caused a reduction of only 2 log units (D_10_ = 0.47 kGy) (28).

The response of the foodborne contaminant *Staphylococcus aureus* towards low-dose gamma-rays as source of ionizing radiation was investigated as a decontamination technology for food preservation (29). Therefore, frozen ham and cheese sandwiches were inoculated with *S. aureus.* Irradiation resulted in an average D_10_ value of 0.625 kGy, indicating that a dose of approximately 3 kGy would result in a 5 log reduction of *S. aureus* in sandwiches.

*B subtilis* and *B. cereus* spores were reduced to approximately 2 log units when a 10 MeV circular electron accelerator was used with 7.6 kGy (30). The corresponding D_10_ values were in the range of 1 to 4 kGy, which was in good accordance with data from gamma irradiation experiments. It has to be pointed out, that in the study of De Lara and coworkers the environmental conditions, like the used type of growth medium, influenced the irradiation efficiency. A previous LEEI study demonstrated, that spore inactivation efficiency was dependent on different external factors, e.g. the sporulation medium used (31). Generally, bacterial spores are more resistant to irradiation than vegetative cells, except for a few very highly radiation-tolerant vegetative bacteria (31). For vegetative *Bacillus* species in suspension, as used in this LEEI study, there are hardly any data on their radiation sensitivity. 32 summarized some D_10_ values for vegetative *B. cereus* which were in the range of 0.30 to 0.65 kGy in phosphate buffer and 0.575 kGy on nutrition broth. The calculated D_10_ values from the *B. subtilis* used here ranged from 0.31 kGy to 0.37 kGy and the inactivation tests were only partially reproducible. This might indicate the presence of a sporulating sub-population. Similar to the environmental influences, the physiological state of the microorganisms, like their different bacterial growth stages, has a great impact on the subsequent LEEI results. *B. pumilus* spores have been widely used as biological indicator to proof the effect of gamma irradiation sterilization process (31, 33, 34). However, inactivation of *Bacillus* spores suspended in water with high-energy accelerated electrons was shown to be less effective than gamma irradiation (24). Summarizing, this excludes them from practical use as a bio-dosimeter for LEEI processes.

The underlying assumption is that ionizing radiation causes the inactivation of microorganisms by losing their ability to multiply. The mechanisms of radiation sensitivity are not yet completely understood and probably vary between different taxa, species, and even strains. Tolerance mechanisms can include effective DNA repair, compatible solutes, and mechanism to detoxify reactive oxygen species, as well as additional strategies to withstand unpredictable environmental changes (35). Radiation susceptibility of microorganisms is additionally affected by different factors such as medium composition, physiological state of the culture, temperature, gas atmosphere, and pH (32). The data set given in **Figure 4** reveals that growth inhibition by LEEI can reproducibly achieved under defined growth and irradiation conditions at room temperature and in air. However, it also points out a certain response variability of each bacterial species towards ionizing radiation.

### Impedance Spectroscopy

Impedance spectroscopy is a rapid and non-invasive method to detect bacterial cells by measuring the change of the electric conductivity. In this study, impedance was used to investigate the response of bacteria to irradiation with low-energy accelerated electrons. *E. coli*, *S. warneri*, and *B. subtilis* showed a delay in growth and the exponential growth phase was entered more slowly with increasing dose. The capability to multiply was affected by an increasing LEEI dose and less surviving (or non-multiplying) bacterial cells remained. This finding was very pronounced in *E. coli* and *S. warneri*, but less evident in *B. subtilis*. Irradiation of *E. coli* and *S. warneri* with 2.8 kGy lead to complete inactivation by inhibition of growth. These results were in good accordance with the inactivation dose experimentally determined by the investigation of the inactivation curves (2.8 kGy). For *B subtilis* growth inhibition was incomplete, which is in accordance with the results from the inactivation kinetics. Although vegetative cells were utilized, a sporulating sub-population may be present, which could explain the inconsistency in determining the inactivation dose.

Impedance spectroscopy has been performed for various applications in microbiology to monitor growth or responses towards environmental changes. To the best of our knowledge, this is the first time that impedance spectroscopy has been successfully used as a method to describe the bacterial response towards irradiation with low-energy accelerated electrons. Whether the irradiated and inactivated bacterial cells have lost their ability to divide, but still show metabolic activity, is yet to be clarified. To investigate the influence of LEEI on the biochemical pathways and gene expression, advanced molecular studies need to be conducted.

## Conclusion

The aim of the study was to verify the suitability of bacterial suspension as bio-dosimeter for LEEI at a dose below 6.5 kGy. The research results demonstrate that LEEI has the potential to replicable inactivate bacteria at doses below 6.5 kGy. The D_10_ values for *E. coli* and *S. warneri* were determined. However, under the given experimental conditions, vegetative cells of *B. subtilis* were not consistent in their response. Therefore, the applicability as reliable dose indicator is currently still uncertain. A workflow standardization in terms of cultivation and irradiation could overcome these hurdles. The bio-dosimeter could be a promising tool to monitor dynamic LEEI processes within fluids, e.g. LEEI-supported biotechnological processes within a bioreactor.

## Data Availability Statement

The original contributions presented in the study are included in the article. Further inquiries can be directed to the corresponding author.

## Author Contributions

SS, GG, and UK designed the study. MD, SG, and LK performed the experiments. SS, GG, and UK interpreted the data. SS wrote the manuscript. All authors contributed to the article and approved the submitted version.

## Conflict of Interest

The authors declare that the research was conducted in the absence of any commercial or financial relationships that could be construed as a potential conflict of interest.

## Publisher’s Note

All claims expressed in this article are solely those of the authors and do not necessarily represent those of their affiliated organizations, or those of the publisher, the editors and the reviewers. Any product that may be evaluated in this article, or claim that may be made by its manufacturer, is not guaranteed or endorsed by the publisher.
